# Experimental Study on Solidification and Stabilization of Heavy-Metal-Contaminated Soil Using Cementitious Materials

**DOI:** 10.3390/ma14174999

**Published:** 2021-09-01

**Authors:** Xiaojun Li, Ruizhi Yang, Hao Li, Hao Yi, Hongjun Jing

**Affiliations:** Xi’an University of Science and Technology, Xi’an 710054, China; y18700962207@126.com (R.Y.); lihao201605@163.com (H.L.); l554748566@163.com (H.Y.); jinghongjun@xust.edu.cn (H.J.)

**Keywords:** heavy-metal-contaminated soil, new curing agent, mechanical properties, leaching characteristics, cementitious materials

## Abstract

In order to solve the shortcomings of the traditional curing agent in the treatment of composite heavy-metal-contaminated soil with the solidification and stabilization method, a new type of cementing material A was used as a curing agent, and the Pb, Cd, Cu composite heavy-metal-contaminated soil was artificially prepared to carry out an experimental study on solidification and stabilization (SS) restoration by the mechanical properties test, leaching performance test, and microscopic test. The results show that in the range of test dosage, with the increase in the curing agent content, the unconfined compressive strength of the solidified body increased, and the resistance to deformation was enhanced. From the perspective of leaching characteristics, the new curing agent A had an excellent curing effect on the composite heavy-metal-contaminated soil. To achieve safe disposal, a curing agent content of 10% applies only for the soil heavily contaminated by heavy metals. The curing agent A could significantly reduce the content of acid-extractable heavy metals after solidifying the heavy metal Pb, Cd, and Cu composite contaminated soil and effectively converted it into a residue state. The solidified phase contained hydrated products such as calcium silicate hydrate (CSH) and ettringite (AFt). These hydrated products can inhibit the leaching performance of heavy metal ions through adsorption, encapsulation, and ion exchange. The study provides a feasible method and reference for the solidification, restoration, and resource utilization of heavy-metal-contaminated soil in the subgrade.

## 1. Introduction

The characteristics of long-term concealment, accumulation and irreversibility make it difficult to control heavy metal pollution in the land, and the environmental risks are prominent [1]. Heavy-metal-contaminated land will cause harm to the ecological environment and human health. At the same time, heavy metals will degrade the engineering mechanical properties of the soil, resulting in cracking of the building structure or settlement and deformation of the foundation [2]. Therefore, the remediation and treatment of soil contaminated by heavy metals are urgent. The traditional methods for the disposal of heavy-metal-contaminated soil are mainly on-site leaching, desorption, electrical methods [3], etc. In recent years, with its lower cost, longer-term stability and mechanical properties, and higher biodegradability, etc., the solidification/stabilization (S/S) method has been widely used in the remediation and treatment of radioactive hazardous waste and heavy-metal-contaminated sites [2,4,5].

The remediation mechanisms of common curing agents of heavy-metal-contaminated soil mainly include adsorption, precipitation, ion exchange, complexation, and oxidation-reduction [3,6,7,8]. Different types of soils contaminated by heavy metals require different types of curing agents, and different types of curing agents have different mechanisms of action. Commonly used curing/stabilizing agents mainly include the following types: curing of inorganic materials such as cement, lime, and fly ash; curing of thermoplastic organic materials such as asphalt and polyethylene; curing of thermosetting organic materials such as urea-formaldehyde and polyester; vitrification technology; stabilizing agents such as ferrous sulfate, phosphate, sodium hydroxide, and polymer organics [2,9,10]. For technical and cost reasons, cement, lime, fly ash and other inorganic materials are widely used as additives for solidification/stabilization [5,11,12]. 

In general, a lot of work has been carried out on the remediation of heavy-metal-contaminated soil both at home and abroad. However, research on the serious heavy metal compound pollution problem is conducted less frequently, and for the heavy metal leaching concentration of the solidified body after solidification, it is difficult to meet requirements or the solidification rate is lower. Therefore, this paper intends to solve the problem through a new type of cementitious material. Through artificial simulation of heavy-metal-contaminated soil, a study on S/S repair test was carried out using a new curing agent A. The mechanical properties and leaching performance of the solidified body specimens were tested. The mechanical properties, deformation characteristics and leaching safety of the solidified body were analyzed on a micro level. Additionally, the changes in the internal composition and structure of heavy-metal-contaminated soil before and after solidification were also observed and analyzed. The study provides technical support for the application of solidified and stabilized heavy-metal-contaminated soil in the subgrade.

## 2. Materials and Methods

### 2.1. Experiment Material

**Figure 1:**
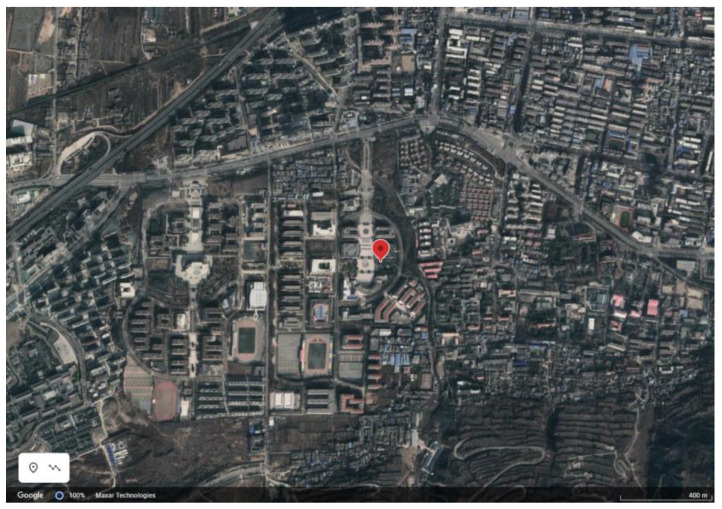
The sampling location (note: the used map was supported by Google Earth^TM^).

The soil to be tested was taken from Lintong District, Xi’an City (the sampling location is shown in Figure 1), and the soil sample was collected at a depth of 30–50 cm below the ground surface. After the sample was naturally air-dried, the plant roots, gravel and other foreign matter were removed. The basic physical and mechanical physicochemical properties of the soil samples are shown in Table 1.

The chemical reagents used in the test are solid lead nitrate, cadmium nitrate, and copper nitrate. The relevant specifications are shown in Table 2.

### 2.2. Sample Preparation

**Figure 2:**
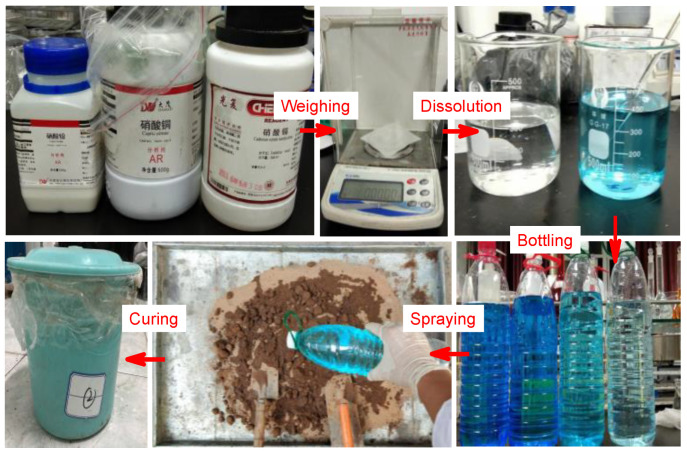
Manual preparation process of soil samples contaminated by heavy metals.

Referring to the soil quality standard [13], the pollution level of the experimental heavy-metal-contaminated soil is classified as pollution-free, light pollution, moderate pollution, and heavy pollution. The concentration of each metal ion is shown in Table 3. The soil sample was naturally air-dried and sieved through the aperture of 2 mm. After the heavy metal solution was configured, the setting concentration of Pb(NO_3_)_2_, Cd(NO_3_)_2_, Cu(NO_3_)_2_ solution were uniformly sprayed into the soil to make the soil sample reach the pre-set concentration. After deionized water was added, the soil sample was stirred, sealed and cured for 30 days under standard curing conditions until the heavy metal ions and the soil were mixed thoroughly to make heavy-metal-contaminated soil. A certain mass of contaminated soil was crushed, and sieved through the aperture of 2 mm to obtain contaminated soil with a particle size of less than 2 mm. The contaminated soil with a particle size of less than 0.5 mm was prepared using the same method. The soil sample flow is shown in Figure 2.

**Figure 3:**
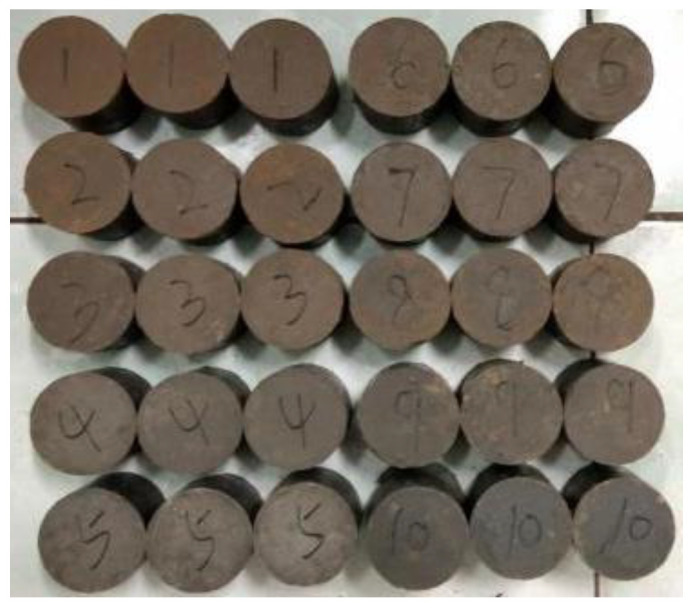
Cured specimen.

In this test, five curing agent content levels were selected; 5%, 10%, 20%, 30%, and 40%, respectively. First, the prepared contaminated soil sample was fully mixed with the curing agent A according to the ratio determined by the plan and deionized water, respectively, with moisture content of 13%. After being fully stirred in a mixer, the sample was formed immediately to prevent moisture volatilization using single pressing molding method. The solidified body sample was a cylindrical sample with a size of φ50 × 50 mm, and three parallel samples were made in each group of tests. With the help of a mold stripping machine the pressed sample was demolded immediately. After that, the sample was wrapped with a water-retaining film and kept in a curing box for 7 days under 60 °C water-bath thermal curing or standard curing. The two groups of cured sample are shown in Figure 3.

The mechanical properties, deformation characteristics and leaching characteristics of the solidified body specimens were tested after being cured. The effects of the dosage of curing agent and the initial pollution concentration on the properties of the solidified body were analyzed, and the minimum dosage of the new curing agent A required for the resource utilization of heavy-metal-contaminated soil was determined.

### 2.3. Experiment Method

#### 2.3.1. Unconfined Compressive Strength Test

**Figure 4:**
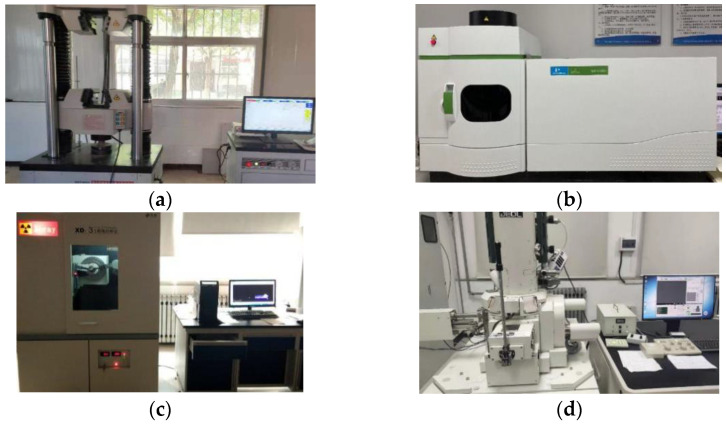
Experimental equipment: (**a**) Unconfined compressive strength tester (Shanghai Bairoe Test Instrument Co., Ltd., Shanghai, China), (**b**) Inductively coupled plasma mass spectrometer (PerkinElmer Optima 8000, Waltham, MA, USA); (**c**) X-ray diffractometer Beijing Puxi General Instrument Co., Ltd, Beijing, China); (**d**) Scanning electron microscope (JSM-T300, Toyoko, Japan).

The unconfined compressive strength test was carried out in accordance with the specification “Test Regulations for Inorganic Binder Stabilized Materials for Highway Engineering” (JTG E51-2009), and the WDW-20 universal testing machine (Shanghai Bairoe Test Instrument Co., Ltd., Shanghai, China) was used for the test. The loading rate during the test was controlled to 2 mm/min. Three parallel samples were tested in each group of experiments, and the results were averaged. The unconfined compressive strength test is shown in Figure 4a.

#### 2.3.2. Toxicity Leaching Test

This test quantitatively evaluated the heavy metal leaching characteristics of the solidified body. The leaching test was carried out in accordance with Chinese environmental protection standard “Solid Waste Toxicity Leaching Method Sulfuric Acid Nitric Acid Method” (HJ/T300-2007). First, 50–100 g samples were selected to test its water content. The crushed sample particles could pass through a 9.5 mm sieve. Then, 75 g samples were put into a 1 L extraction bottle. According to the water content of the sample and the ratio of liquid to solid of 10:1, the required amount of extractant was calculated (concentrated sulfuric acid: concentrated nitric acid = 2:1) and added into the bottle, which was put in a flip oscillation device for 24 h, with the bottle cap tight. Finally, the filter membrane was installed on the pressure filter to filter and collect leachate, and the concentration of the leachate was examined by the inductively coupled plasma mass spectrometer (ICP-MS) (PerkinElmer Optima 8000, PerkinElmer, Waltham, MA, USA). The test process is shown in Figure 4b.

#### 2.3.3. Contaminated Soil Sample Heavy Metal Form Test

**Figure 5:**
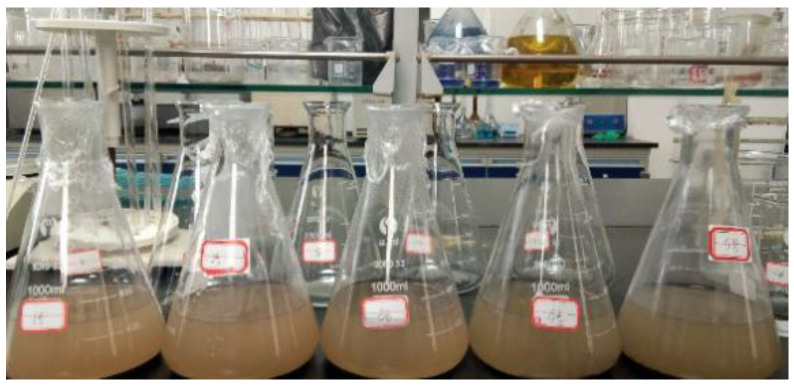
The extraction process of heavy metal morphology.

The BCR method was used to determine the content of available heavy metals in contaminated soil samples. The heavy metal elements in the soil usually exist in exchangeable state, carbonate-bound state, Fe-Mn oxide-bound state, organic-bound state and residual state. The experiment process is shown in Figure 5. The specific extraction steps are as follows:

(1) Adding 1 mol/L MgCl2 solution to the selected samples and adjusting pH to 7.0, oscillating continuously for 1 h at 25 °C, and centrifuging for 20 min. The supernatant was taken and diluted to 25 mL as the exchangeable solution to be tested.
(2) Adding 16 mL of 1 mol/L NaAc solution to the residue in the previous step, adjusting pH to 5.0, followed by continuous oscillation at 25 °C for 8 h. After centrifugation for 20 min, the supernatant was taken and diluted to 25 mL as the carbonate-bound solution.
(3) Adding 16 mL of 25% HAC solution of 0.04 mol/L NH_2_OH HCl to the residue in the previous step, oscillating intermittently at 96 °C for 4 h and centrifuging for 20 min. The supernatant was taken and diluted to 25 mL as the Fe-Mn oxide bound solution.
(4) Adding 3 mL of 0.01 mol/L HNO_3_ and 5 mL of 30% H_2_O_2_ to the residue, adjusting the pH value to 2.0, water-bath heating to 85 °C for 2 h and cooling to 25 °C. Then, 5 mL of 20% HNO_3_ solution of 3.2 mol/L NH_4_Ac was diluted to 20 mL, oscillated for 30 min and centrifuged for 20 min. The supernatant was taken out and diluted to 25 mL as the organic bound solution.
(5) Taking out the residue in the previous step and completely digesting with graphite digester. The solution was canceled as the residual state to be measured.

The solution to be tested and digestion solution extracted in the above five steps were filtered, and the concentrations of Pb, Cd and Cu ions in each step were measured by ICP-MS.

#### 2.3.4. X-ray Diffraction (XRD)

X-ray diffraction test is used to analyze the phase of the heavy-metal-contaminated soil before and after solidification. In the test, the powder of the crushed sample which could pass through a 300-mesh sieve was placed in the slot of a glass slide. After the sample flattened and compacted, the slide was placed on the loading table, and the corresponding diffraction intensity at 2θ was collected by X-ray emission and with a certain speed. The crystal content and structure were determined by the diffraction intensity. The higher peak value indicates that the crystal structure was more complete and contained more material components [14,15,16].

The uncured heavy-metal-contaminated soil used in the XRD test is the heavily contaminated soil, while the solidified body is heavily contaminated soil with 20% curing agent. The X-ray diffractometer (XD-3 automatic X-ray powder diffractometer, Beijing Puxi General Instrument Co., Ltd., Beijing, China) used in this experiment is shown in Figure 4c. The related parameters of the experiment are: fixed copper target, tube voltage 36 kV, tube current 20 mA, scanning starting angle 10°, ending angle 70°, step scanning 5°/min, step width 0.02°.

#### 2.3.5. Scanning Electron Microscope Test (SEM-EDS)

SEM/EDS tests were conducted to observe the microstructure and mineral composition of soil samples contaminated by heavy metals before and after solidification. Before the test, the soil sample was dried, broken and sieved through 100 mesh, and then the sample powder was sprinkled on the conductive adhesive and adhered to the sample tray table. The sample was sprayed with gold within 12 h before scanning. After that, the processed sample was placed on the sample table, vacuumed, and the image was scanned. The image magnification is 5000 times, and the results obtained by scanning include SEM microstructure images, distribution maps of various elements obtained by EDS, and relevant information of point energy spectrum. The scanning electron microscope equipment (JSM-T300, JEOL, Japan) used in this experiment is shown in Figure 4d.

## 3. Test Results and Analysis

### 3.1. Analysis of the Mechanical Properties of the Cured Body

#### 3.1.1. The Effect of Curing Agent Content on Mechanical Properties

**Figure 6:**
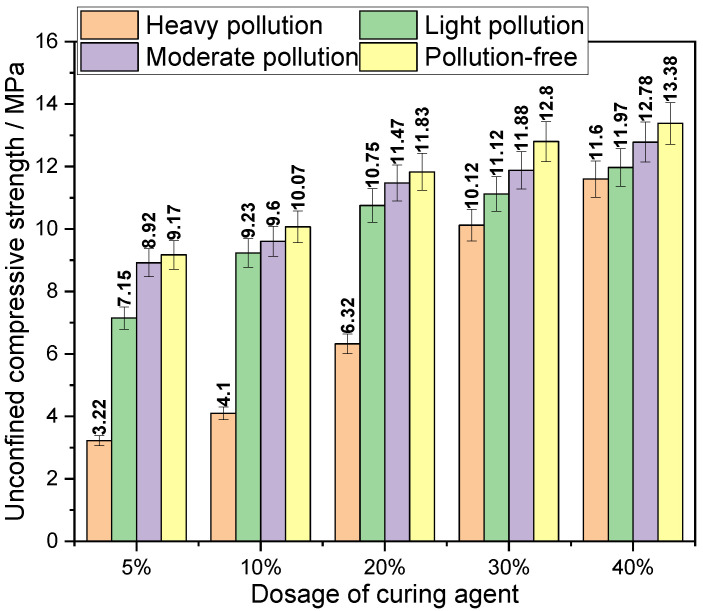
Compressive strength of solidified contaminated soil with different curing agent content.

The variation in unconfined compressive strength of the solidified body with the amount of curing agent is shown in Figure 6. An experimental study on the relationship between the unconfined compressive strength of the solidified body and the amount of curing agent under four initial pollution degrees was carried out.

As is shown in Figure 6, the relationship between the intensity and the amount of curing agent added can be concluded under the same initial pollution degree of heavy metal Pb, Cd, and Cu composite contaminated soil, that is, with the amount of curing agent increasing from 5% to 40%, the histogram is constantly rising, indicating that with the increase in the curing agent content, the unconfined compressive strength of the solidified body also increases, and the trend of the contaminated soil solidified body with different initial pollution degrees is consistent. The unconfined compressive strength can reach up to 13.38 MPa. This may be because, on the one hand, the new curing agent A is a small particle component, which can change the particle gradation of heavy-metal-contaminated soil macroscopically and make the overall structure dense. On the other hand, the formation of the whole strength of the curing agent and the contaminated soil is a long-term and complex process. The curing agent and the heavy-metal-contaminated soil are mixed and reacted by hydration and hydrolysis to form a whole with a cementing structure with higher strength. From a microscopic point of view, the main products of cement, fly ash, and silica fume in the curing agent after hydration are ettringite (AFT), calcium silicate hydrate (CSH), calcium aluminate hydrate (CAH), etc. [17] With the further hydration of the curing agent material, the amount of hydration products continues to increase to form the intertwined crystalline packages on the surface of the soil particles, which play the role of bonding and filling, and increase the strength of the contaminated soil.

It can be seen from Figure 6 that for the soil samples with the same initial pollution degree, when the dosage of curing agent increases from 5% to 40%, the increasing ranges of unconfined compressive strength of the solidified body are 25–41%, 39–57%, 18–60% and 4–14%, respectively. It can be found that after the curing agent content exceeds 30%, the unconfined compressive strength increases significantly with the increase in curing agent content. This may be due to the fact that too much curing agent material is added and cannot be fully involved in the hydration and hydrolysis reaction. As a result, a considerable part of the curing agent does not play its role. Therefore, simply adding more curing agent to improve the mechanical properties of the solidified body may not achieve the expected effect.

#### 3.1.2. Influence of Initial Pollution Degree on Mechanical Strength

**Figure 7:**
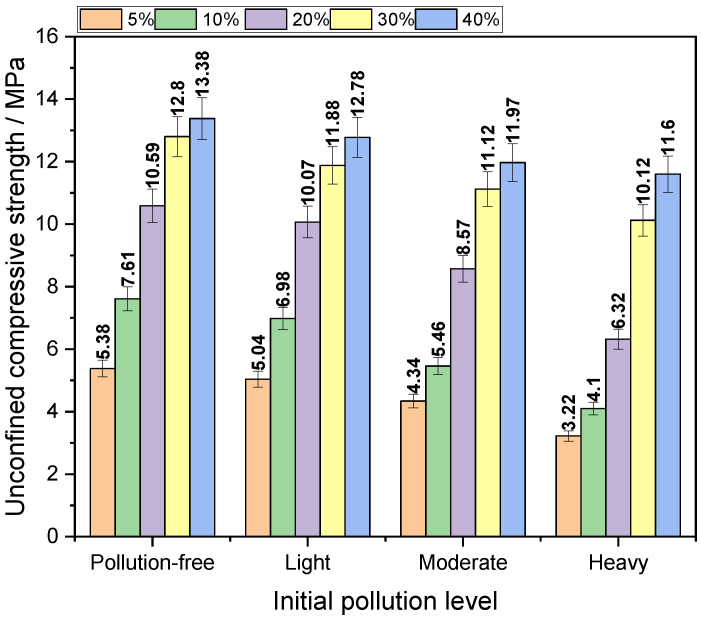
Compressive strength of solidified contaminated soil under different initial pollution degree.

The variation in unconfined compressive strength of the solidified body with the initial pollution degree is shown in Figure 7. An experimental study on the relationship between the unconfined compressive strength of the solidified body and the initial pollution degree under the dosage of five curing agents was carried out.

In Figure 7, the relationship between the strength and the initial pollution degree of the heavy metal Pb, Cd, and Cu composite contaminated soil with the same dosage of curing agent can be obtained, that is, with the initial pollution degree rising from non-pollution to heavy pollution, the histogram is constantly decreasing, which shows that with the aggravation of the initial pollution degree, the unconfined compressive strength of the solidified body decreases, and the trend of solidified form of contaminated soil with different dosage of curing agent is consistent.

For the heavily polluted soil samples, the amount of curing agent increases from 5% to 40%, and the unconfined strength of the solidified body increases by 260%. When the dosage of curing agent is 20%, the unconfined strength is 6.32 MPa, which is higher than the geological landfill standard (5 MPa). When the content of curing agent is 30%, the unconfined strength is 10.12 mpa, which meets the requirements of building material strength (10 MPa). For moderately contaminated soil samples, the content of curing agent increases from 5% to 40%, and the unconfined strength of the cured body increases by 175%. When the content of curing agent is 10%, the unconfined strength is 5.42 MPa, which is higher than the geological landfill standard. According to the standard, when the curing agent content is 30%, the unconfined strength is 11.32 MPa, which meets the strength requirements of building materials. For lightly contaminated soil samples, the content of curing agent increases from 5% to 40%, and the unconfined strength of the cured body increases by 153%. When the content of curing agent is 5%, the unconfined strength is 5.04 MPa, which is higher than the standard. When the curing agent content is 20%, the unconfined strength is 10.07 MPa, which meets the strength requirements of building materials.

In summary, as the amount of curing agent increases, the unconfined compressive strength of the solidified body also increases, and the trend of solidified body of the contaminated soil with different initial pollution degrees is consistent. The maximum unconfined compressive strength of the solidified body can reach 13.38 MPa. After the curing agent content exceeds 30%, the unconfined compressive strength will decrease significantly with the increase in curing agent content. Simply adding more curing agent to improve the mechanical properties of the solidified body may not achieve the desired effect. In terms of mechanical properties alone, for heavily polluted soil samples, the content of curing agent must reach 20% to achieve safe disposal. To meet the strength requirements of building materials, the content of curing agent must reach 30%. For moderately contaminated soil samples, the content of curing agent must reach 10% to achieve the purpose of safe disposal, and to meet the strength requirements of building materials, the content of curing agent must also reach 30%. For lightly contaminated soils, the content of curing agent needs to reach 5% to achieve the purpose of safe disposal. To meet the strength requirements of building materials, however, the content of curing agent also needs to reach 20%.

### 3.2. Analysis of the Deformation Characteristics of the Solidified Body

**Figure 8:**
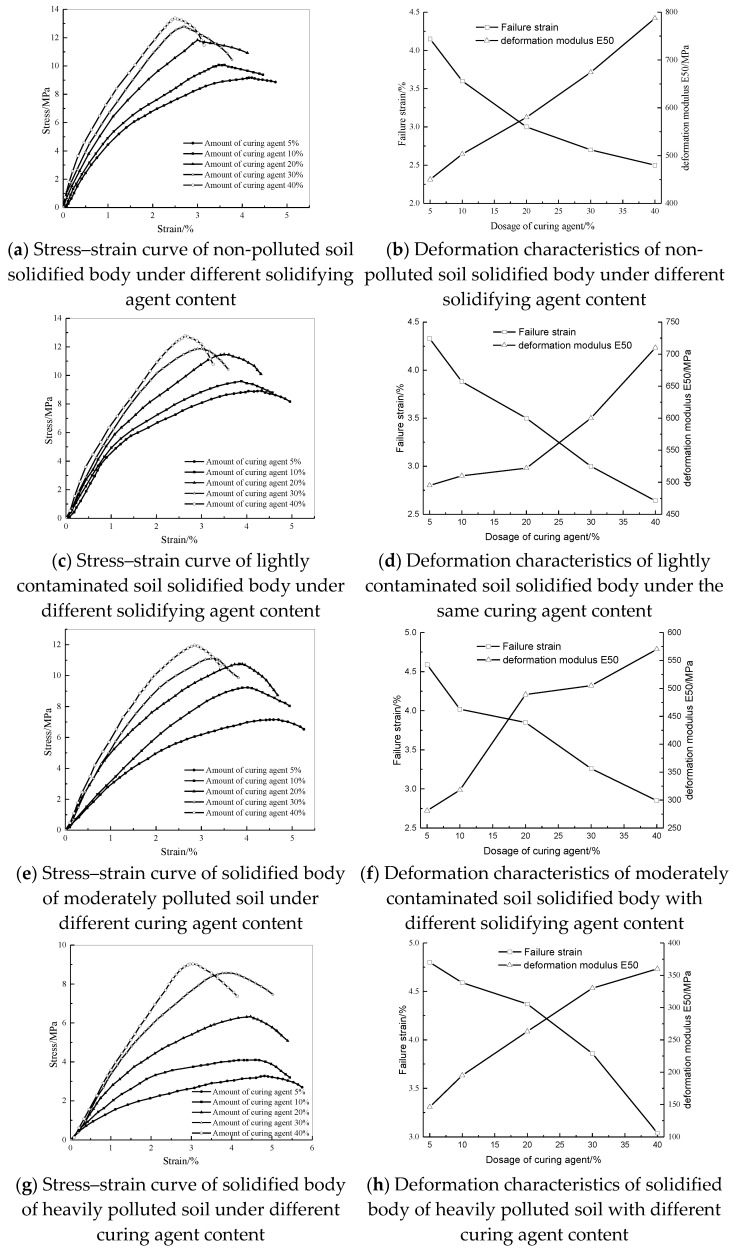
The deformation law of the cured body under different curing agent content.

Since the solidified body of heavy-metal-contaminated soil is nonlinearly deformed, the deformation modulus is not a constant. The deformation modulus E_50_ is usually used to characterize the deformation characteristics of the material. E_50_ refers to the secant modulus corresponding to 50% of the peak stress, also known as deformation coefficient [3]. Therefore, according to the stress–strain curve, the failure strain and the deformation modulus E_50_ are obtained to analyze the deformation characteristics of the solidified body of heavy-metal-contaminated soil. Figure 8 shows the stress–strain curve, failure strain curve, and deformation modulus of the solidified body of different heavy metal pollution levels with different curing agent content.

It can be seen from Figure 8 that the change trend of stress–strain curves of different initial contamination degree is similar under different dosage of curing agent. If the dosage of curing agent is low (5% and 10%), the peak value of stress–strain curve is not obvious. If the dosage of curing agent is high (20%, 30% and 40%), the stress increases obviously with strain at the beginning of compression, and there is a significant peak after reaching the ultimate strength. After the peak, the structure of the solidified body is destroyed, and the stress–strain curve decreases rapidly. With the increase in the amount of curing agent, the stress–strain curve shows a peak left-up shift, which represents the weakening of the plasticity of the solidified body and the enhancement of the resistance to deformation.

It can be seen from the regular curves of curing agent dosage, failure strain and deformation modulus E_50_ in Figure 8 that for solidified bodies of different initial pollution degrees, with the increase in curing agent dosage, the failure strain of solidified body gradually decreases, the peak value in the corresponding stress–strain curve shifts to the left. Moreover, with the increase in curing agent dosage, the extent of the reduced failure strain gradually decreases, and the corresponding curve of the relationship between the failure strain and the dosage of curing agent tends to flatten out gradually. It can be seen from the relationship curve between the deformation modulus E_50_ and the dosage of curing agent that with the increase in the dosage of curing agent, the deformation modulus E_50_ has a significant nonlinear increasing trend. When the content of curing agent increases from 5% to 10%, the increasing range of E_50_ is the largest. With the further increase in the content of curing agent, the increasing range of E_50_ gradually decreases.

### 3.3. Analysis on Leaching Characteristics of Solidified Form

**Figure 9:**
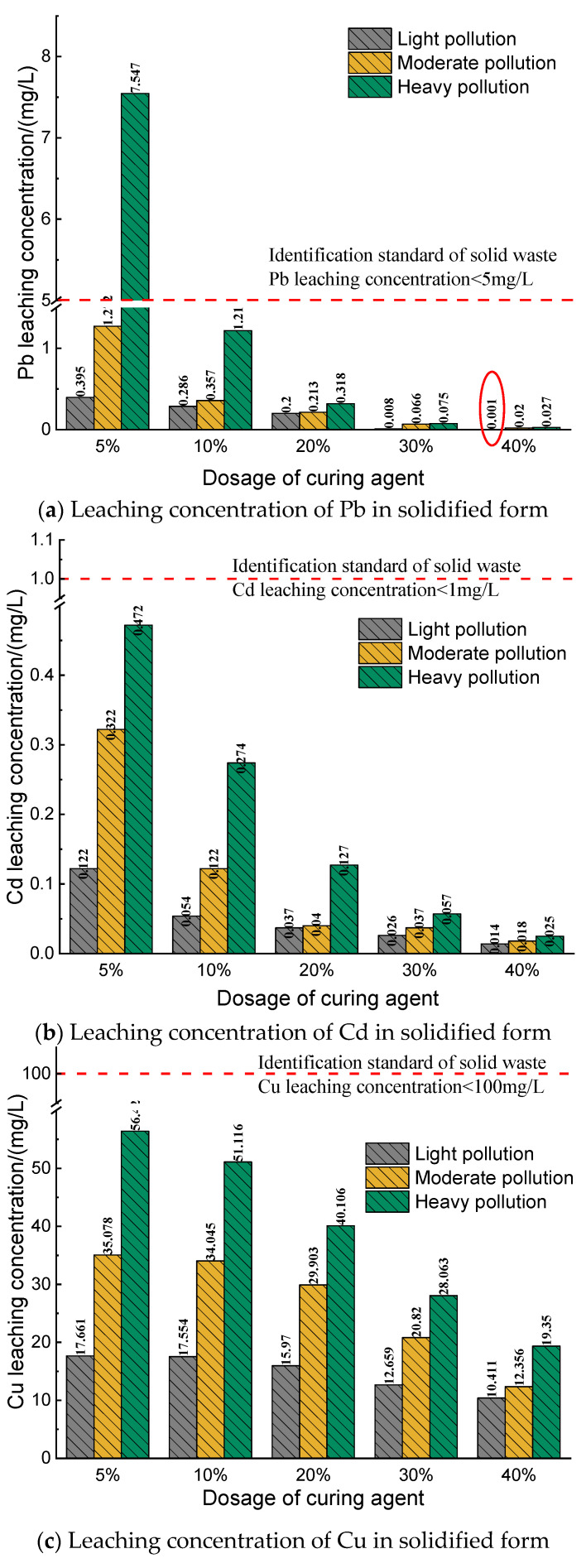
The leaching concentration of heavy metals in the solidified body.

The leaching concentration diagram of heavy metals Pb, Cd, and Cu in the solidified body is shown in Figure 9. According to the “Identification Standard for Hazardous Wastes Leaching Toxicity Identification” (GB 5085.3-2007), the safety standard limit for Pb leaching is 5 mg/L, the safety standard limit for Cd leaching is 1 mg/L, and the safety standard limit for Cu leaching is 100 mg/L. 

It can be seen from Figure 9a that after the heavy-metal-contaminated soils of three pollution degrees are solidified with different dosages of curing agent and cured for 7 days, the leaching concentrations of Pb in the solidified body are mostly lower than the safety limit. The leaching concentration of heavily polluted soil is 7.547 mg/L when the curing agent content is 5%, which is slightly higher than the standard safety limit for leaching. The leaching concentration of Pb in all other solidified bodies is lower than the limit. Additionally, the Pb leaching concentration of lightly polluted soil solidified by 40% curing agent is lower than the minimum detection limit of equipment 0.001 mg/L, and it was expressed as 0.001 when the data were collated. It can be seen from Figure 9 that with the increase in the curing agent content, the leaching Pb concentration decreases significantly, and the leaching concentration of Pb in lightly and moderately contaminated soil solidified by 5% curing agent is lower than the safety limit. The leaching concentration of Pb in three kinds of contaminated soil solidified by 10% curing agent is lower than the safety limit. After the curing agent content reaches 20%, the leaching concentration of Pb drops significantly, which is far below the safety limit.

It can be seen from Figure 9b that after the heavy-metal-contaminated soils of three pollution degrees are solidified with different dosages of curing agent and cured for 7 days, the leaching concentrations of Cd in the solidified body are all lower than the safety limit of 1 mg/L. Similar to the leaching concentration of Pb in the solidified body, the leaching concentration of Cd in the solidified body decreases significantly with the increase in the amount of curing agent. For the heavily polluted soil, when the content of curing agent increases from 5% to 40%, the leaching concentration of Cd decreases by 94.7%, which is far below the leaching safety standard limit.

It can be seen from Figure 9c that after the heavy-metal-contaminated soils of three pollution degrees are solidified with different dosages of curing agent and cured for 7 days, the leaching concentrations of Cu in the solidified body are all lower than the safety limit of 100 mg/L. Although the leaching concentration of Cu in the contaminated soil solidified by 5% curing agent is lower than the leaching safety standard limit, the leaching concentration continues to decrease with the increase in the curing agent content. However, with the increase in the curing agent content, the leaching concentration of Cu does not decrease as obviously as that of Pb and Cd, especially in lightly polluted soil. When the content of curing agent increases from 5% to 20%, leaching concentration of Cu decreases to only 9.5%.

In summary, it can be seen from the analysis of the leaching performance test results of the optimized solidified soil that with the increase in the amount of curing agent, the leaching concentrations of three heavy metals Pb, Cd and Cu in the solidified soil also decrease, and the trend of solidified soil with different initial pollution degrees is consistent. Only in heavily polluted soil, the leaching concentration of Pb slightly exceeds the limit after leaching test of the solidified body with 5% curing agent, and the leaching concentrations of Cd and Cu in the same group reach the standard. The leaching concentrations of three heavy metals Pb, Cd and Cu decrease with the increase in the amount of curing agent. The decrease in Pb and Cd is relatively significant, while Cu decreases slightly. The leaching concentration of Pb in slightly and moderately polluted soils is lower than the safety limit if the amount of the curing agent is 5% and the leaching concentrations of Pb in three polluted soils are all lower than the safety limit if the dosage is 10%. As for Cd and Cu, the leaching concentrations of the three contaminated soils after solidification are all lower than the safety limit with curing agent of 5%. Therefore, in terms of leaching safety of the solidified body, 10% curing agent is required for the heavily polluted soil to achieve safe disposal, whereas for other contaminated soils, the dosage of curing agent of 5% can be satisfying.

### 3.4. Microstructure Analysis of Solidified Body

**Figure 10:**
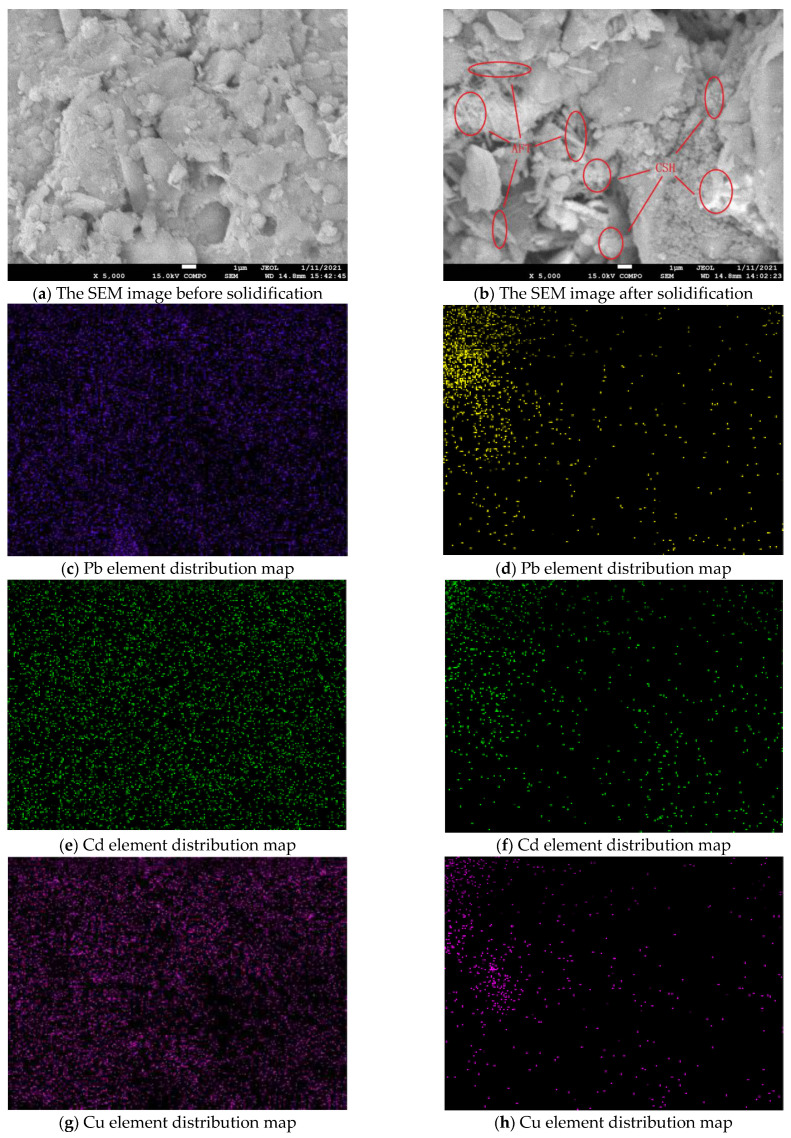

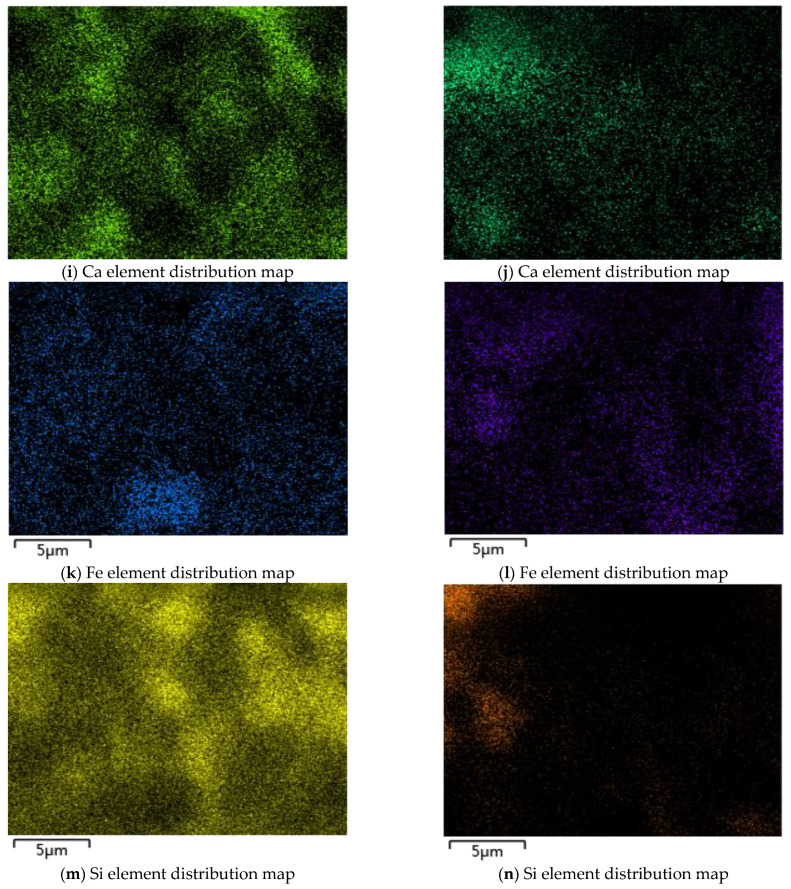
Microscopic morphology and distribution of main elements of contaminated soil before and after solidification.

The SEM/EDS diagram and distribution diagram of main elements (including lead, cadmium, copper, calcium, iron and silicon) of heavy-metal-contaminated soil before solidification are shown in Figure 10. The content of Pb, Cd and Cu in the heavy-metal-contaminated soil samples were 10,000 mg/kg, 40 mg/kg and 6000 mg/kg, respectively. Among a large number of scanning images, the images with a magnification of 5000 times were selected to observe its micro morphology. It can be seen from its microstructure distribution that the internal structure of the contaminated soil sample is loose, the particles are small and dispersed obviously. The bonding characteristics between the soil particles are damaged to a certain extent, and the whole structure is relatively loose, which may be due to the addition of heavy metals to change and destroy the original structure of the soil particles.

In addition, it can be seen from the element distribution diagram that the distribution of heavy metals Pb, Cd, and Cu is significantly different from that of Ca, Fe, and Si. The distribution of elements Pb, Cd, and Cu are distributed throughout the entire image, and the distribution is uniform, without obvious regularity. The distribution of elements Ca, Fe, and Si has obvious regularity, and is related to the distribution of particles. The shape and edge of the distribution are consistent with the shape and edge of the soil particles. It can also be seen that the heavy metals Pb, Cd, Cu have no obvious dependence on mineral elements.

The SEM/EDS map and the distribution map of main elements (including lead, cadmium, copper, calcium, iron, and silicon) after solidification of heavy-metal-contaminated soil are shown in Figure 10. The contents of Pb, Cd, and Cu of heavy-metal-contaminated soil samples artificially prepared in the experiment were 10,000 mg/kg, 40 mg/kg and 6000 mg/kg, respectively, with 20% curing agent. On the basis of a large number of scanning images, the images with a magnification of 5000 times were finally selected to observe its microscopic appearance. It can be seen from the microstructure distribution map that a large number of needle-like substances and flocculent substances appear on the microscopic image of the soil sample contaminated by heavy metals after solidification. A large number of previous studies have shown that this type of substance is the hydration product of the curing agent material in an alkaline environment [18,19,20,21]. Among them, needle-like material is ettringite (AFT), and flocculation material is mostly calcium silicate hydrate (CSH) gel. In addition, some structures similar to AFT or CSH gel hydrated products can also be detected, but there are some differences in structure and morphology. The distribution of heavy metals Pb, Cd and Cu is significantly different from that before solidification. It can be inferred that the structure changes after the hydration products absorb heavy metals and it is this kind of structure that plays the role of solidifying heavy metals.

### 3.5. Morphology Analysis of Solidified Metal

**Figure 11:**
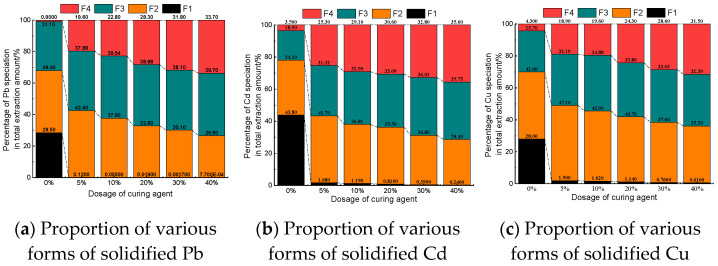
Proportion of heavy metals in solidified form.

After solidification treatment and curing for 7 days, the occurrence forms of Pb, CD and Cu in the solidified body are shown in Figure 11. 

In the uncured original soil samples, heavy metal Pb mainly presents in the form of F1 (weak acid state), F2 (reducible state) and F3 (oxidizable state) with contents of 28.5%, 39.5%, and 31.1%, respectively. F4 (residual state) is extremely low, accounting for 0.9%. After solidification, the main forms of Pb in the contaminated soil are F2 (reducible), F3 (oxidizable) and F4 (residual). In addition, with the increase in the amount of curing agent, the content of F1 (weak acid state) gradually decreases, whereas the content of F2 (reducible state) does not change much, and the overall trend is gradually decreasing. The content of F3 (oxidizable state) also changes little, but the overall trend is gradually increasing. The content of F4 (residual state) increases greatly, which is 36 times higher than that without curing. This shows that with the increase in solidified agent content, Pb in contaminated soil transforms to F3 (oxidizable state) and F4 (residual state) and tends to be stable.

In uncured original soil samples, Cd mainly presents in the form of F1 (weak acid state) and F2 (reducible state) with contents of 43.8% and 34.2%. The content of F3 (oxidizable state) is 18.5% and F4 (residual state), 3.5%, is very low. After solidification, the main forms of Cd in contaminated soil are F2 (reducible), F3 (oxidizable) and F4 (residual). In addition, with the addition of curing agent, the content of F1 (weak acid state) decreases rapidly, the content of F4 (residual state) increases significantly, and the contents of F2 (reducible state) and F3 (oxidizable state) change little. With the increase in the dosage of curing agent, the content of F1 (weak acid state) constantly decreases. When the dosage of curing agent increases from 5% to 40%, the content of F1 (weak acid state) decreases by 85%. The content of F4 (residual state) increases with the increase in the dosage of curing agent. When the dosage of curing agent increases from 5% to 40%, the content of F4 (residual state) increases by 40.7%. This indicates that the addition of the curing agent can transform a considerable proportion of active heavy metal Cd in contaminated soil into a relatively stable form.

In the uncured original soil samples, Cu mainly presents in the form of F1 (weak acid state), F2 (reducible state)/F3 (oxidizable state) with contents of 28%, 42%, and 25.7%, respectively. F4 (residual state) is relatively low; 4.3%. After solidification, the main forms of Cu in the contaminated soil are F2 (reducible), F3 (oxidizable) and F4 (residual). In addition, with the addition of curing agent, the content of F1 (weak acid state) decreases rapidly, the content of F4 (residual state) increases significantly, and the contents of F2 (reducible state) and F3 (oxidizable state) change little. With the increase in the content of curing agent, the content of F1 (weak acid state) decreases. When the content of curing agent increases from 5% to 40%, the content of F1 (weak acid state) decreases by 67.9%; The content of F4 (residual state) increases with the increase in curing agent dosage. When the dosage of curing agent increases from 5% to 40%, the content of F4 (residual state) increases by 66.7%, and the contents of F2 (reducible state) and F3 (oxidizable state) change little. However, with the increase in curing agent dosage, F2 (reducible state) decreases while F3 (oxidizable state) increases. This indicates that the addition of curing agent can transform a considerable proportion of active heavy metal Cu in contaminated soil into F3 (oxidizable state) and F4 (residual state) and tend to be stable.

To sum up, it can be seen that the new curing agent A can significantly reduce the content of the weak acid-extractable heavy metals after solidification of Pb, Cd and Cu composite contaminated soil, and effectively transform it into residual state. Increasing the amount of curing agent can further reduce the content of weak acid extraction in the solidified body and improve the content of its residual state. Meanwhile, the content of oxidizable state decreases slightly and the content of reducible state increases slightly.

### 3.6. Solid Phase Analysis

**Figure 12:**
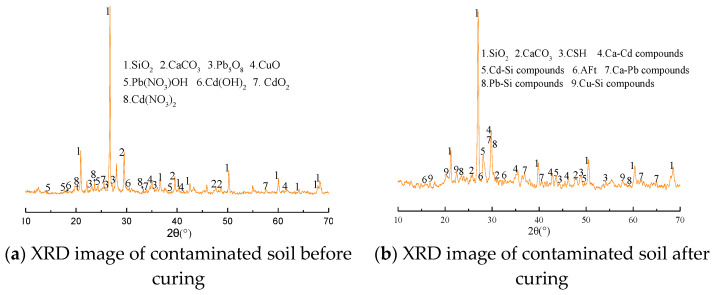
XRD images.

The phase analysis is a qualitative analysis based on the processing of the XRD spectrum. It can determine the mineral composition according to the diffraction angle and peak value of the mineral [22,23]. The contaminated soils combined with heavy metals Pb, Cd, and Cu before and after solidification were selected for X-ray diffraction test. The test results are shown in Figure 12.

**Figure 13:**
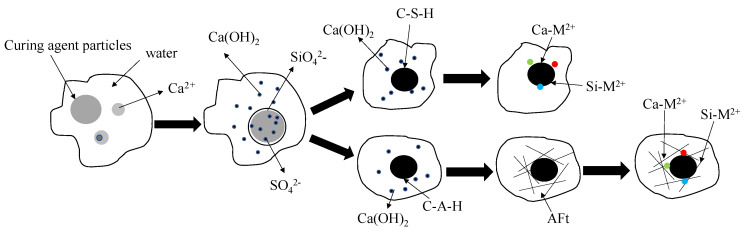
Schematic diagram of new phase formation principle.

It can be seen from Figure 12 that SiO_2_ and CaCO_3_ are the main minerals in the heavy-metal-contaminated soil before solidification, and Pb_5_O_8_, CdO_2_ and CuO of Pb, Cd and Cu as well as other compounds Pb(NO_3_)OH, Cd(OH)_2_ and Cd(NO_3_)_2_ of Pb, Cd and Cu can be detected in the secondary minerals. Figure 12b shows the XRD image of the solidified heavy-metal-contaminated soil. The figure also shows that the internal material composition of the heavy-metal-contaminated soil changes after solidification by the new curing agent, and some heavy metal compounds disappear due to the addition of the curing agent. At the same time, new phases are retrieved, mainly including hydrated calcium silicate hydrate (CSH), ettringite (Aft) and other stable compounds combined with heavy metals and Ca and Si ions. The principle of the formation process of the new phases is shown in Figure 13.

New phases such as hydrated calcium silicate (CSH) and ettringite (AFT) can encapsulate the heavy metal ions. Some of them will undergo ion exchange or addition and combine with calcium and silicon ions to from a stable structure, so that lead, cadmium and copper ions can be stabilized in the contaminated soil. When the diffraction angle is 29.4°, the diffraction peak before solidification is CaCO_3_, and after solidification, the single peak is searched at the same diffraction peak position. It is found that there are a variety of Ca-Cd compounds, Cd-Si compounds and Pb-Si compounds with extreme similarity. It is determined that there should be a stable compound formed by the adsorption and encapsulation of calcium and silicon products produced by the hydration of curing agent on heavy metal ions.

## 4. Conclusions

In this work, a new type of cementing material was used as a curing agent, and the Pb, Cd, Cu composite heavy-metal-contaminated soil was artificially prepared to carry out experimental study on solidification, stabilized restoration. The mechanical properties, deformation characteristics and leaching safety of the solidified body were analyzed based on the mechanical properties test, leaching performance test, and microscopic test of the solidified body specimens. The following main conclusions may be drawn from the results of the present study:

(1) The mechanical properties of the contaminated soil solidified by the new curing agent A are superior, and the maximum unconfined compressive strength is 33.45 MPa, while the minimum is 3.22 MPa. However, simply adding more curing agent to improve the mechanical properties of the solidified body may not achieve the desired effect.
(2) With the increase in the amount of curing agent, the resistance to deformation of solidified soil is significantly enhanced, which is manifested as the decrease in failure strain and the increase in the deformation modulus E_50_ under compressive load, and the trend of solidified soil with different initial pollution degree is consistent.
(3) From the aspect of leaching characteristics, the new curing agent has an excellent solidifying effect on the composite heavy-metal-contaminated soil. To achieve the purpose of safe disposal, the dosage of curing agent should reach 10%, which only applies for the heavily polluted soil. For other soils of different pollution degrees, 5% of the dosage is satisfying. This study, however, only discusses the properties of solidified polluted soil which were cured for 7 days, and its long-term environmental safety and mechanical properties need to be further studied.
(4) The content of weak acid-extractable heavy metals can be significantly reduced and effectively transformed into residual state by the new curing agent A. Increasing the amount of curing agent can further reduce the content of weak acid extraction form and improve the content of residual form. After solidification, there are hydrated products such as calcium silicate hydrate (CSH) and ettringite (AFT), which can inhibit the leaching performance of heavy metal ions by adsorption, encapsulation and ion exchange.

## Figures and Tables

**Table 1:** Basic physical and chemical properties of test soil.

Natural Moisture Content *w*/%	Natural Density *ρ*/(g/cm^3^)	Plastic Limit *w_p_*/%	Liquid Limit *w_L_*/%	Cohesion *c*/kPa
16.3	1.67	17.4	37.2	60

**Table 2:** Test chemical reagent information table.

Reagent Name	Chemical Formula	Manufacturer	Purity
Lead nitrate	Pb(NO_3_)_2_	Tianjin Beilian Fine Chemicals Development Co., Ltd.	Analytically pure
Cadmium nitrate	Cd(NO_3_)_2_·4H_2_O	Tianjin Guangfu Fine Chemical Research Institute	Analytically pure
Copper nitrate	Cu(NO_3_)_2_·3H_2_O	Tianjin Damao Chemical Reagent Factory	Analytically pure

**Table 3:** Optimization test of heavy metal level settings in compound contaminated soil.

Heavy Metal Pollution Level	Heavy Metal Content/(mg·kg^−1^)		
	Pb	Cd	Cu
Pollution-free	0	0	0
Light pollution	2000	8	1200
Moderate pollution	6000	24	3600
Heavy pollution	10,000	40	6000
